# Return-to-Work Status Following One- and Two-Level Anterior Cervical Discectomy and Fusions: A Prospective Cohort Study

**DOI:** 10.7759/cureus.27546

**Published:** 2022-08-01

**Authors:** Elham Mirzamohammadi, Negar Qasemian, Negin Kassiri, Saber Mohammadi, Jaber Hatam, Hasan Ghandhari

**Affiliations:** 1 Occupational Medicine Department, School of Medicine, Iran University of Medical Sciences, Tehran, IRN; 2 Department of Neurosurgery, Hazrat Rasool Akram Hospital, Iran University of Medical Sciences, Tehran, IRN; 3 Bone and Joint Reconstruction Research, Shafa Orthopedic Hospital, Iran University of Medical Sciences, Tehran, IRN

**Keywords:** absenteeism, return to work, cervical discectomy, cervial spine, cervical disc

## Abstract

Background: The purpose of this article was to determine the rate of return to work (RTW) and contributing factors after a one- and two-level anterior cervical discectomy and fusion (ACDF), a common spine surgery. Recognizing the contributing factors to RTW of occupationally active patients is important.

Methodology: In this study, 68 patients were examined at three, six, and nine months after ACDF by the same team and same spinal surgeon at a single medical center, and the rate of RTW and contributing factors were determined. In this study, relationships were analyzed by the logistic regression method.

Results: The results of this study demonstrated that 77.9%, 82.4%, and 82.4% of workers had returned to work after three, six, and nine months, respectively. At nine months, 82.4% of the patients had returned to work, 19.6% returned to part-time work, and 80.4% had returned to their previous work. Conversely, 17.6% of the patients had not returned to work after nine months. In the logistic regression analysis, older age, longer absence from work before surgery, and less employer support were the related factors for no RTW.

Conclusions: Per the results, it may be concluded that nearly 82% of patients with ACDF had returned to work after nine months of follow-up. Lack of RTW is affected by older age, longer absence from work before surgery, and employer support. Planning according to these variables can reduce the burden of the problem.

## Introduction

Cervical spondylosis is a relatively common musculoskeletal disorder that results from cervical disc degeneration and causes neck pain, especially in middle-aged and elderly patients, which causes functional limitations [1]. Factors other than aging that can cause cervical spondylosis include neck injury, work-related activities that put extra strain on the neck such as the lifting of heavy objects, holding the neck in an awkward position for a long time, recurrent neck movements throughout the day (causing repetitive pressure), genetic factors such as a family history of cervical spondylosis, smoking history, being overweight, and a sedentary lifestyle. Due to the radiculopathy and myelopathy which can accompany this disorder, the patient develops progressive signs and symptoms that need treatment due to these numerous problems and reduced quality of life [2,3]. This disorder, which is seen in 15% of the general population, is treated either medically or surgically depending on the patient's condition [4]. Since surgical treatments are limited to only specific patients, we need to plan for conservative and non-invasive treatments such as medications, physiotherapy, acupuncture, and some alternative medicines, especially for the group of patients in their early stages of the disease [5].

Since the results after surgical treatments vary according to the fusion methods used in different countries [6], there is a need for detailed studies on patients undergoing various methods to assess the postoperative conditions such as the rate of return to work (RTW). Failure to RTW after an illness in a person who was previously productive can have great economic and social costs for the individual and the society [7]. Parameters such as psychosocial factors, fear, avoidance of returning to work, and employee-employer relationships are effective in returning to work [8]. Sometimes other non-physical factors such as patient motivation, job satisfaction, and benefits also affect the patient's decision to RTW more than the physical factors and barriers such as the need for high physical ability in the workplace [8-11]. Previous studies in different countries reported the RTW percentages after anterior cervical discectomy and fusions (ACDFs) to be between 48% and 89.3% by studying patients with different conditions [12-15]. In developing countries, due to economic problems caused by unemployment, the rate of RTW is expected to be higher. To date, however, few studies have been conducted in this field. Identifying the factors influencing RTW in patients who have been previously active is of particular importance. Accordingly, in this study, we examined the RTW and the factors affecting it after one- and two-level ACDF.

## Materials and methods

In this study, which was performed as a prospective cohort study, all patients who underwent ACDFs between 2020 and 2022 in one of the training hospitals of the Iran University of Medical Sciences were selected. Inclusion criteria were the patients being employed for the year prior to surgery and having their characteristics available for follow-up. Exclusion criteria included age < 18 years and > 75 years; a history of previous neck surgery; myelopathy having an origin other than spondylitis such as trauma, tumor, infection, and neurovascular; or a deficiency of the information in the file, which could not be corrected by a phone call to the patient. Initially, demographic and medical information questionnaires were completed by each patient who was an ACDF surgical candidate, and they included the patient's age at the time of admission, gender, dates of their hospitalization, job title, work experience, monthly income, marital status, education, regular exercise (> three times a week and >30 minutes each time), shift work (working other than routine working hours), and the number of ACDF level. In addition, by using the neck disability index (NDI) questionnaire, the patient’s function was determined. The NDI questionnaire is a tool for the detection of neck-specific disability and has 10 items concerning pain intensity and activities of daily living including personal care, concentration, reading, headaches, lifting, work status, driving, sleeping, and recreation activities. To calculate the NDI, since each of the 10 NDI items is given a number of points from 0 to 5, we divided the total scores obtained from the NDI questionnaire by 50 and obtained a percentage. The validity and reliability of the Persian version of the NDI questionnaire were confirmed by a study by Mousavi et al., which had a Cronbach's alpha coefficient of 0.88 [16]. In calculating the disability based on the NDI questionnaire, we included patients with a percentage of disability less than or equal to 30% in one group and those with a percentage of disability more than 30% in the second group [17]. Using the visual analysis scale (VAS), the amount of neck pain and radicular pain in the upper extremities in a range of 0 with the highest level of pain as 10 was determined.

All patients with spondylosis and radiculopathy, suffering from neck pain radiating to the upper extremities and decreased grip force, were candidates for an ACDF. The ACDF procedure was used for all patients and was performed by the same spine surgery group in a single medical center. During the postoperative follow-up visits at intervals of three, six, and nine months, RTW was obtained from each patient through a checklist containing the variables of non-working days, the reason for leaving work, first full-time working day, type of insurance, employer and co-worker support, job satisfaction, and time of RTW after nine months. In the case of no face-to-face visit, RTW information was obtained by phone calls. To assess insurance, employer and co-worker support, each individually, and job satisfaction, we asked patients to assign a number from one to 10, representing the lowest and highest levels of support and job satisfaction, respectively. The job title was determined at the time of hospitalization and after returning to work, and was assigned into one of the two categories of white- and blue-collar work. Next, all demographic and socioeconomic variables between individuals who did and did not RTW were compared, and the items that showed significance in univariate analysis were examined by multivariate logistic regression analysis. The effective factors in returning to work were identified. In all stages of the study, the principles of medical ethics were observed in accordance with the Helsinki Treaty. Admission to the study was voluntary. Patients were not charged for any part of the project. Patient privacy was strictly upheld. If it was necessary to call people, in cases where the person was not willing to answer questions, the conversation was discontinued. Before starting work, the code of ethics IR.IUMS.FMD.REC.1399.212 was obtained on 2020-06-21.

Statistical analysis

Data were analyzed with the Statistical Product and Service Solutions (SPSS) software version 24 (IBM Corp., Armonk, NY). Mean and standard deviation indices were used to describe the quantitative variables, frequency number, and the percentage of qualitative variables. A T-test was performed to compare the quantitative variables, and a chi-square test was used to compare the qualitative variables.

## Results

In this study, 68 patients who underwent ACDFs in one of the training hospitals of the Iran University of Medical Sciences between 2020 and 2022 were included. Demographic, occupational, and disease-related characteristics of individuals were as follows: 54.5% of the study population were male, 87% were married, and 63% were nonsmokers. Approximately, 73% of the population had a high school degree or completed a portion of their education. Only 17.6% of people mentioned a history of regular exercise > three times a week and > 30 minutes each time. More than two-thirds of the population was in the blue-collar occupational classification; 86% of the population was covered by insurance and 31% by supplementary insurance. A total of 73.5% of patients reported pain referred to the upper extremities, and 58.8% of patients mentioned a history of comorbidity. Hypertension with a 35% prevalence rate was the most common comorbidity in the subjects followed by musculoskeletal diseases (30%) and diabetes (25%). None of the subjects in the study received rehabilitation treatment after surgery. The average age of the subjects was 46.53 years with an average work experience of 20.75 years, and the average working hours per week was 44.41 hours. Descriptive analysis of other quantitative variables is presented in Table 1.

**Table 1:** Descriptive analysis of quantitative variables *A scale of 1 to 10 was assigned by the patient with 10 being the highest level of job satisfaction. **A visual analog scale of 1 to 10 was assigned by the patient with 10 being the highest level of pain. ***A scale of 1 to 10 was assigned by the patient with 10 being the highest level of support. BMI: Body mass index; NDI: Neck disability index.

Variables	Mean ± standard deviation	Minimum	Maximum
Age	48.53 ± 9.4	22	70
Number of children	2.22 ± 1.4	0	4
Body mass index	26.18 ± 3.6	18.25	34.72
Work experience (years)	20.75 ± 10.6	3	52
Time of work per week (hours)	44.41 ± 13.9	8	84
Time of shift work (hours)	18.75 ± 5.5	7	24
Job satisfaction*	7.32 ± 1.8	3	10
Duration of neck pain (months)	20.93 ± 16.1	1	60
The amount of neck pain**	6.84 ± 1.5	2	9
The amount of upper extremity pain	5.25 ± 2.7	0	9
NDI score (%)	45.52% ± 17.9%	10%	90%
Duration of hospitalization (days)	2.01 ± 0.4	0	3
Duration of sickness (days)	14 ± 0.0	14	14
Duration of absence from work before surgery (days)	26 ± 25.7	1	90
Employer support***	7.68 ± 1.7	3	10
Co-worker support***	8.03 ± 1.3	5	10
Insurance support***	7.08 ± 1.7	3	10

In this study, out of 68 patients, 12 (17.6%) had not returned to work and 56 (82.4%) had returned to work. Of these 56, 10 (17.8%) had chosen new jobs (white-collar) due to disability and 46 (82.4%) had returned to their previous jobs. Of the 56 people who had returned to work, 45 (80.4%) were employed full-time and 11 (19.6%) were employed part-time. The rate of RTW at three, six, and nine months after surgery were 77.9%, 82.4%, and 82.4%, respectively.

We divided the patients into two groups: those who had returned to work and those who had not returned to work and examined the relationship between RTW and quantitative and qualitative variables (Tables 2, 3). In a univariate analysis based on the chi-square test, RTW showed a statistically significant association with having supplementary insurance, a white-collar primary job, and no history of comorbidity (p-value < 0.05). According to the independent T-test, RTW was statistically significantly associated with lower mean age, shorter working hours per week, shorter duration of neck pain, more employer support, more co-worker support, and less absenteeism before surgery (p-value < 0.05).

**Table 2:** Univariate analysis of RTW and qualitative variables * > Three times a week and >30 minutes each time. **Working other than routine working hours. RTW: Return to work; NDI: Neck disability index.

		RTW	No RTW	P-values
Gender	Male	31 (83.8%)	6 (16.2%)	0.73
	Female	25 (80.6%)	6 (19.4%)	
Marital status	Married	47 (79.7%)	12 (20.3%)	0.13
	Single	9 (100%)	0 (0%)	
Smoking history	Yes	24 (96%)	1 (4%)	0.02
	No	32 (74.4%)	11 (25.6%)	
Regular exercise history*	Yes	12 (100%)	0 (0%)	0.07
	No	44 (76.6%)	12 (21.4%)	
Primary job	White collar	19 (100%)	0 (0%)	0.01
	Blue collar	37 (75.5%)	12 (24.5%)	
Shift work**	No	48 (80%)	12(20%)	0.16
	Yes	8 (100%)	0 (0%)	
Insurance	Yes	48 (81.4%)	11(18.6%)	0.58
	No	8 (88.9%)	1 (11.1%)	
Supplementary insurance	Yes	21 (100%)	0 (0%)	0.01
	No	35 (74.5%)	12 (25.5%)	
Upper extremity pain	No	14 (77.8%)	4 (22.2%)	0.55
	Yes	42 (84%)	8 (16%)	
Comorbidity	No	28 (100%)	0 (0%)	0.001
	Yes	28 (70%)	12 (30%)	
Number of ACDF levels	One	33 (84.6%)	6 (15.4%)	0.57
	Two	23 (79.3%)	6 (20.7%)	
NDI score (%)	≤30%	12 (100%)	0 (0%)	0.07
	>30%	44 (78.6%)	12 (21.4%)	

**Table 3:** Univariate analysis of return to work and quantitative variables *A scale of 1 to 10 was assigned by the patient with 10 being the highest level of job satisfaction. **A visual analog scale of 1 to 10 was assigned by the patient with 10 being the highest level of pain. ***A scale of 1 to 10 was assigned by the patient with 10 being the highest level of support. RTW: Return to work; BMI: Body mass index; NDI: Neck disability index.

		Mean ± standard deviation	P-values
Age	RTW	44.98 ± 9.0	0.003
	No RTW	53.75 ± 7.7	
Body mass index	RTW	25.83 ± 3.6	0.08
	No RTW	27.81 ± 2.9	
Smoking history (packs/year)	RTW	6.11 ± 3.2	0.48
	No RTW	3.75 ± 0	
Work experience	RTW	19.88 ± 10.8	0.14
	No RTW	24.83 ± 9.2	
Working hours per week	RTW	42.50 ± 13.3	0.014
	No RTW	53.33 ± 14.2	
Job satisfaction*	RTW	7.52 ± 1.8	0.058
	No RTW	6.42 ± 1.7	
Duration of neck pain (months)	RTW	16.95 ± 13.2	0.000
	No RTW	39.50 ± 15.8	
Neck pain	RTW	6.84 ± 1.7	0.99
	No RTW	6.83 ± 0.7	
Upper extremity pain**	RTW	5.20 ± 2.6	0.23
	No RTW	5.50 ± 3.4	
NDI score (%)	RTW	21.89 ± 8.8	0.08
	No RTW	26.83 ± 9.0	
Hospitalization (days)	RTW	2.00 ± 0.5	0.58
	No RTW	2.08 ± 0.2	
Absenteeism before surgery (days)	RTW	21.07 ± 20.5	0.000
	No RTW	49.00 ± 35.8	
Employer support***	RTW	7.95 ± 1.5	0.003
	No RTW	6.33 ± 1.9	
Coworkers support***	RTW	8.23 ± 1.3	0.007
	No RTW	7.08 ± 1.2	
Insurance support***	RTW	7.20 ± 1.7	0.27
	No RTW	6.58 ± 1.5	

We categorized patients into two groups as follows and examined the relationship between RTW and quantitative and qualitative variables: Group 1 consisted of patients returning to the previous job and Group 2 were patients who did not return to a previous job (including returning to a new job or not returning to work after nine months).

A univariate analysis based on the chi-square test, return to previous job with female gender (p-value: 0.03), a one-level ACDF (p-value: 0.001), being single (p-value: 0.02), white-collar job (p-value: 0.000), work shift history (p-value: 0.03), history of regular exercise as defined earlier (p-value: 0.008), absence of comorbidity (p-value: 0.000), and less than 30% disability per the NDI score (p-value: 0.008) showed a significant association. Based on the independent T-test, return to previous work showed a significant association with lower mean age (p-value: 0.001), less work experience (p-value: 0.001), shorter neck pain duration (p-value: 0.000), lower NDI score (p-value: 0.004), and shorter absence from preoperative work (p-value: 0.005).

We then entered the cases that were significant in the univariate analysis into the logistic regression test, and it was found that RTW was statistically significantly related to the variables of age, employer support, and length of absence from work before surgery (p-value < 0.05). Also, return to previous work was significantly associated with age, the number of ACDF levels, and the NDI score (p-value < 0.05).

## Discussion

Cervical spondylosis is a relatively common musculoskeletal disorder that causes intermittent neck pain, functional limitations, and reduced quality of life [1]. Different factors affect the RTW [7]. Identifying these factors in patients who have been previously active is especially important.

In this study, the rates of RTW at three, six, and nine months after surgery were 77.9%, 82.4%, and 82.4%, respectively. According to the logistic regression test, RTW was statistically significantly related to the variables of age, employer support, and length of absence from work before surgery (p-value < 0.05). Also, return to previous work was significantly associated with age, the number of discectomy levels, and NDI score (p-value < 0.05).

Lied et al. in a cohort study in Norway on 258 patients undergoing ACDF found that the rate of RTW after six months was 48%, and the success rate was 78% [12]. In our study, the rate of RTW after six months was slightly higher (82.4%). In a cohort study by Devin et al. in the United States, it was clarified that the rate of RTW during the three-month period was 86.2%. Factors associated with their reduced RTW included excessive absenteeism from preoperative work, older age, part-time preoperative employment, having heavy jobs, receiving compensation for disability, and higher severity of cervical involvement [13]. In our study, the rate of RTW after three months was less (77.9%). We reported factors that were associated with a reduction in RTW as older age, less employer support, and longer absenteeism before surgery. Failure to return to previous work had a significant association with older age, higher number of ACDF levels, and higher NDI scores, the latter of which were indicative of higher disease severity and disability. A cohort study by Goh et al. in Singapore on 219 cases of the number of ACDF levels found that the rate of RTW was lower in people with a greater degree of preoperative myelopathy [14]. In our study, the return to previous work was lower in people with higher levels of ACDF and higher NDI scores. The latter two parameters are indicators of higher severity of the disease and disability. Bhandari et al. in a retrospective cohort found that 49 patients undergoing ACDF had a 62% RTW rate. Higher severity of preoperative neck pain, higher postoperative neck pain, and older age were accompanied by a lower RTW; patients' gender and the number of levels of ACDF, however, had no effect on RTW [15]. In our study, the rate of RTW at three, six, and nine months after surgery was 77.9%, 82.4%, and 82.4%, respectively. We also obtained similar results with respect to age and RTW. In our study, unlike the study of Bhandari et al., return to previous work had a significant association with the number of ACDF.

The strength of this study is that all patients were examined and evaluated by one spine surgeon, and spondylosis and radiculopathy diagnoses were confirmed for all of them by that surgeon. In addition, the same ACDF surgical procedure was performed by the same surgical team at only one medical center. Considering several factors affecting the RTW was another strength of the present study. Limitations of this study were incomplete information in the patients' files and access to patients’ contact phone numbers as well as a lack of proper cooperation in the patients' answering of various questions. It was also sometimes difficult for patients to remember accurate information, despite their willingness to cooperate (recall bias). In addition, due to the COVID-19 pandemic, a number of elective surgeries were postponed.

Increasing the sample size, reviewing and following up with patients for a long time, and considering other effective factors in returning to work such as alternative surgical techniques and receiving additional sessions of rehabilitation may add more meaningful connections and are suggested in future studies.

## Conclusions

Based on the results of this study, most of the patients returned to work after six months. Since returning to work after three months was also high, workers can be assured not to worry about returning to work after undergoing ACDF surgery. Older age, less employer support, and longer absenteeism time are the most important factors related to not returning to work after a one- or two-level ADCF. Also, return to previous work was significantly associated with age, the number of ACDF levels, and NDI score. Programming according to these variables can reduce the burden of the problem.
